# Incidence and risk factors of acute coronary syndrome in younger age groups

**DOI:** 10.1186/1865-1380-8-S1-P4

**Published:** 2015-04-22

**Authors:** Abhishek Sharma, Yasser Chomayil, Firoz Abdul Karim, Venugopalan Poovathum Parambil

**Affiliations:** 1 MIMS Kottakkal, Kerala, India

## Objectives

Acute Coronary Syndrome (ACS) is one of the major causes of mortality and morbidity globally. The incidence of ACS in the younger population is rising; hence, there is a need for more careful evaluation and proper disposition of this subset population.

This study determines the incidence, risk factors and mortality of Myocardial Infarction (STEMI & NSTEMI) among hospitalized patients presenting to the ED with chest pain ages 40 years or less.

## Methodology

Patient-records were reviewed and collected from the Medical Record Department and analyzed in terms of variables such as risk factors, type of MI, and mortality.

This was a retrospective study of all patients who presented to the ED and were admitted with a diagnosis of Myocardial Infarction at MIMS Kottakkal over a span of 2 years (August 2012- August 2014). All patients aged 18-40 presenting to ED with a complaint of chest pain / discomfort and admitted with a diagnosis of myocardial infarction were included, and patients with congenital cardiac defects were excluded.

## Results

A total of 955 patients were admitted with Myocardial Infarction in these 2 years, out of which 37 patients (3.9 %) fulfilled the inclusion criteria. The mean age was 36.14 years, Male:Female ratio was found to be 8:1 . Incidence of AWMI, IWMI, NSTEMI and LWMI were 36.2 %, 33.33 %,30.5 % and nil cases respectively. The risk factors in our patients were dyslipidemia, smoking, diabetes and hypertension , & family history.

## Conclusion

Healthcare providers should be vigilant and have a high index of suspicion when dealing with patients presenting with chest pain less than 40 years old than to discharge them as non-cardiac pain without appropriate evaluation and workup.

